# Eight-Year Outcomes of Implantation of Posterior Chamber Phakic Intraocular Lens With a Central Port for Moderate to High Ametropia

**DOI:** 10.3389/fmed.2021.799078

**Published:** 2021-12-16

**Authors:** Kazutaka Kamiya, Kimiya Shimizu, Masahide Takahashi, Wakako Ando, Hideki Hayakawa, Nobuyuki Shoji

**Affiliations:** ^1^Visual Physiology, School of Allied Health Sciences, Kitasato University, Tokyo, Japan; ^2^Department of Ophthalmology, Sanno Hospital, Tokyo, Japan; ^3^Department of Ophthalmology, School of Medicine, Kitasato University, Tokyo, Japan

**Keywords:** EVO-ICL, phakic IOL, long-term prognosis, safety, efficacy, predictability, stability, myopia

## Abstract

**Purpose:** To assess the 8-year clinical outcomes of implantation of an implantable collamer lens (ICL) with a central port (KS-Aquaport; EVO-ICL) for moderate to high myopia and myopic astigmatism.

**Methods:** This retrospective study comprised a total of 177 eyes of 106 patients with spherical equivalents of −7.99 ± 3.33 D [mean ± standard deviation], who underwent EVO-ICL implantation. We evaluated the safety, efficacy, predictability, stability, and adverse events of the surgery, at 1 month, and 1, 2, 4, 6, and 8 years postoperatively.

**Results:** The logarithm of the minimal angle of resolution (LogMAR) uncorrected distance visual acuity (UDVA) and corrected distance visual acuity (CDVA) were −0.07 ± 0.17 and −0.20 ± 0.09, respectively, at 8 years postoperatively. The safety and efficacy indices were 1.18 ± 0.24 and 0.89 ± 0.28, respectively. At 8 years, 83 and 93% eyes were within ± 0.5 D and ± 1.0 D of the targeted correction, respectively. Change in manifest refraction from 1 month to 8 years postoperatively was −0.13 ± 0.30 D. Three eyes (1.7%) that developed cataracts had a slight pre-existing peripheral anterior subcapsular cataract formation required simultaneous ICL extraction and cataract surgery at 2 or 3 years or ICL size change (1 size up) at 7 years postoperatively. We found that neither significant intraocular pressure (IOP) rise (including pupillary block) nor significant endothelial cell loss occurred in any case throughout the 8-year observation period.

**Conclusions:** Current ICL implantation with central port technology offered good continuous outcomes for all measures of safety, efficacy, predictability, and stability for correcting moderate to high myopic errors over a long period, thereby suggesting its long-term viability as a surgical approach for the treatment of such eyes.

## Background

Implantable Collamer Lens (EVO Visian ICL^TM^, STAAR Surgical, Monrovia, CA, USA), a posterior chamber phakic intraocular lens, has been well-recognized as a long-term safe and effective surgery all over the world to correct moderate to high refractive errors (1–6). However, conventional implantable collamer lens (ICL) implantation has several disadvantages over keratorefractive surgeries in the necessity of preoperative laser iridotomy or intraoperative peripheral iridectomy to avoid pupillary block and the possible risk of cataract formation. The EVO ICL with central port technology (KS-AquaPORT V4c and V5; EVO-ICL) was first developed by Shimizu et al. (7, 8) to rectify such drawbacks without significant deterioration in visual performance (9). It has been reported to effectively correct not only moderate to high myopia (10–14) but also low to moderate myopia (15, 16). Moreover, ICL surgery is mainly reversible and allows for ICL exchange, unlike laser in situ keratomileusis (LASIK), even when the patients experience unexpected postoperative vision (17). However, to the best of our knowledge, no long-term studies on current ICL implantation with central port technology have so far been conducted spanning more than 7 years. Long-term study on current ICL implantation with central port technology may give us intrinsic insights into understanding the long-term prognosis of the patients undergoing modern ICL surgery, assuming that postoperative complications such as cataract formation and a significant IOP rise could be greatly reduced by introducing this promising technology. The goal of the present study is to retrospectively and longitudinally evaluate the long-term clinical results of EVO-ICL implantation for the correction of moderate to high ametropia, with particular attention to the late-onset complications. As far as we can ascertain, this study assesses the longest-term (up to 8 years) follow-up outcomes in a cohort of patients undergoing current hole technology-based ICL surgery.

## Materials and Methods

### Study Population

This study protocol was enrolled with the University Hospital Medical Information Network Clinical Trial Registry (000044268). Our study comprised a total of 156 eyes of 106 consecutive patients, who underwent ICL implantation of the current ICL model (EVO-ICL, V4c and V5) at Kitasato University Hospital between January 2007 and June 2017 for the correction of moderate to high myopia and myopic astigmatism, and who completed a 4-year to 8-year follow-up (visited our hospital at least once during the 4 to 8 years postoperatively for routine examinations). The inclusion criteria for ICL surgery at our institution include unsatisfactory correction with spectacles or contact lenses, age between 20 and 50 years at the time of surgery, stable refraction, −3.00 to −14.0 diopters (D) of myopia with astigmatism of 3 D or less, anterior chamber depth (the corneal endothelium to the crystalline lens, ACD) ≥ 2.8 mm, and endothelial cell density (ECD) ≥ 1800 cells/mm^2^. Any history of ocular surgery, corneal diseases, including keratoconus and pellucid marginal degeneration, glaucoma, uveitis, retinal diseases, or other concomitant eye diseases, were excluded from the study. The study was approved by the Institutional Review Board at Kitasato University Hospital (identifier: B21-118) and followed the tenets of the Declaration of Helsinki. Written informed consent for EVO-ICL surgery was obtained from all the patients after explaining the possible consequences.

### Outcomes Measures

Preoperatively and at 1 month, and at 1, 2, 4, 6, and 8 years postoperatively, we assessed the following metrics: the logarithm of the minimal angle of resolution (logMAR) of uncorrected distance visual acuity (UDVA) and corrected distance visual acuity (CDVA), the manifest spherical equivalent (MSE), intraocular pressure (IOP) using a non-contact tonometer, ECD using a non-contact specular microscope, and the vault between the anterior surface of the crystalline lens and the posterior surface of the ICL using an anterior segment optical coherence tomography (AS-OCT) (CASIA, Tomey Corporation Co. Ltd., Aichi, Japan) (from 2017 to 2021), in addition to routine slit-lamp biomicroscopic and funduscopic examinations. All available visit data were collected and grouped according to the closest time point. If more than one visit was available within a given grouping, we utilized the visit data comparable to the given time point for this analysis.

### Power Calculation and Size Selection

We determined the ICL size (12.1, 12.6, 13.2, and 13.7 mm), mainly based on the manufacturer's nomogram, using white-to-white distance, and the ACD using a scanning-slit light corneal tomographer (Orbscan,IIz, Bausch&Lomb, Rochester, USA) or the AS-OCT. We also selected the ICL power using an online calculation and ordering system provided by the manufacturer based on a modified vertex formula (18, 19). We usually selected a toric model ICL in eyes with manifest astigmatism of 1 D or more and a non-toric model ICL in eyes with less than 1 D.

### Surgical Procedures

We described the details of the surgical procedures in our previous studies (10–12, 15). In brief, on the day of the surgery, dilating and topical anesthetic agents were applied. A model V4c or V5 ICL was implanted through a temporal clear corneal incision of 3 to 3.2 mm after injection of a viscosurgical substance into the anterior chamber. Next, the ICL was inserted into the posterior chamber, the viscosurgical substance was replaced with a balanced salt solution, and a miotic agent was administered. We topically used antibiotic and steroidal medications 4 times daily for 1 week, by gradually reducing the dose.

### Statistical Analysis

The normality of all data samples was first checked using the Shapiro–Wilk test. Since all data fulfilled the criteria for normal distribution, the paired and unpaired *t*-test were used to compare the pre- and post-surgical treatment. The one-way analysis of variance (ANOVA) test was used to assess the time-course of changes, with the Dunnett test being employed for multiple comparisons. The Fisher's exact test was used to compare the percentages between the two groups. Unless otherwise indicated, the results are expressed as mean ± standard deviation [95% confidence interval (CI)], and a value of *p* < 0.05 was considered statistically significant.

## Results

### Study Population

A total of 177 eyes (including 79 of men and 98 of women) of 106 patients met the inclusion criteria of this study. Table 1 shows the preoperative baseline demographics of the study population. The mean follow-up period was 6.3 ± 1.7 years (95% CI, 3.0 to 9.6 years). The number of eyes examined at each visit were 177 (100%), 177 (100%), 177 (100%), 177 (100%), 109 (62%), and 60 (34%), respectively, at 1 month, and 1, 2, 4, 6, and 8 years postoperatively.

**Table 1:** Preoperative patient demographics of the study population.

**Parameters**	**Mean ± SD (95%CI)**
Number of eyes	177
Age	35.9 ± 7.9 years (21.2 to 50.6 years)
Male: Female	79: 98
Log UDVA	1.32 ± 0.31 (0.72 to 1.92)
LogMAR CDVA	−0.15 ± 0.09 (−0.34 to 0.03)
Manifest spherical equivalent	−7.99 ± 3.33 D (−14.51 to −1.46 D)
Manifest cylinder	−1.13± 1.20 D (−3.48 to 1.22 D)
Intraocular pressure	13.0 ± 2.5 mmHg (8.0 to 17.9 mmHg)
Mean keratometric readings	43.51 ± 1.83 D (39.92 to 47.11 D)
Anterior chamber depth	3.11 ± 0.29 mm (2.55 to 3.68 mm)
White-to-white distance	11.6 ± 0.4 mm (10.8 to 12.3 mm)
Central corneal thickness	528.7 ± 33.8 μm (462.4 to 594.9 μm)
Axial length	26.97 ± 1.64 mm (23.76 to 30.18 mm)
Toric: Non-toric	76: 101
ICL size 12.1: 12.6: 13.2: 13.7	29: 78: 66: 4

*SD, standard deviation; CI, confidence interval; logMAR, logarithm of the minimal angle of resolution; UDVA, uncorrected distance visual acuity; CDVA, corrected distance visual acuity; D, diopter; ICL, implantable collamer lens*.

### Safety and Efficacy Outcomes

**Figure 1:**
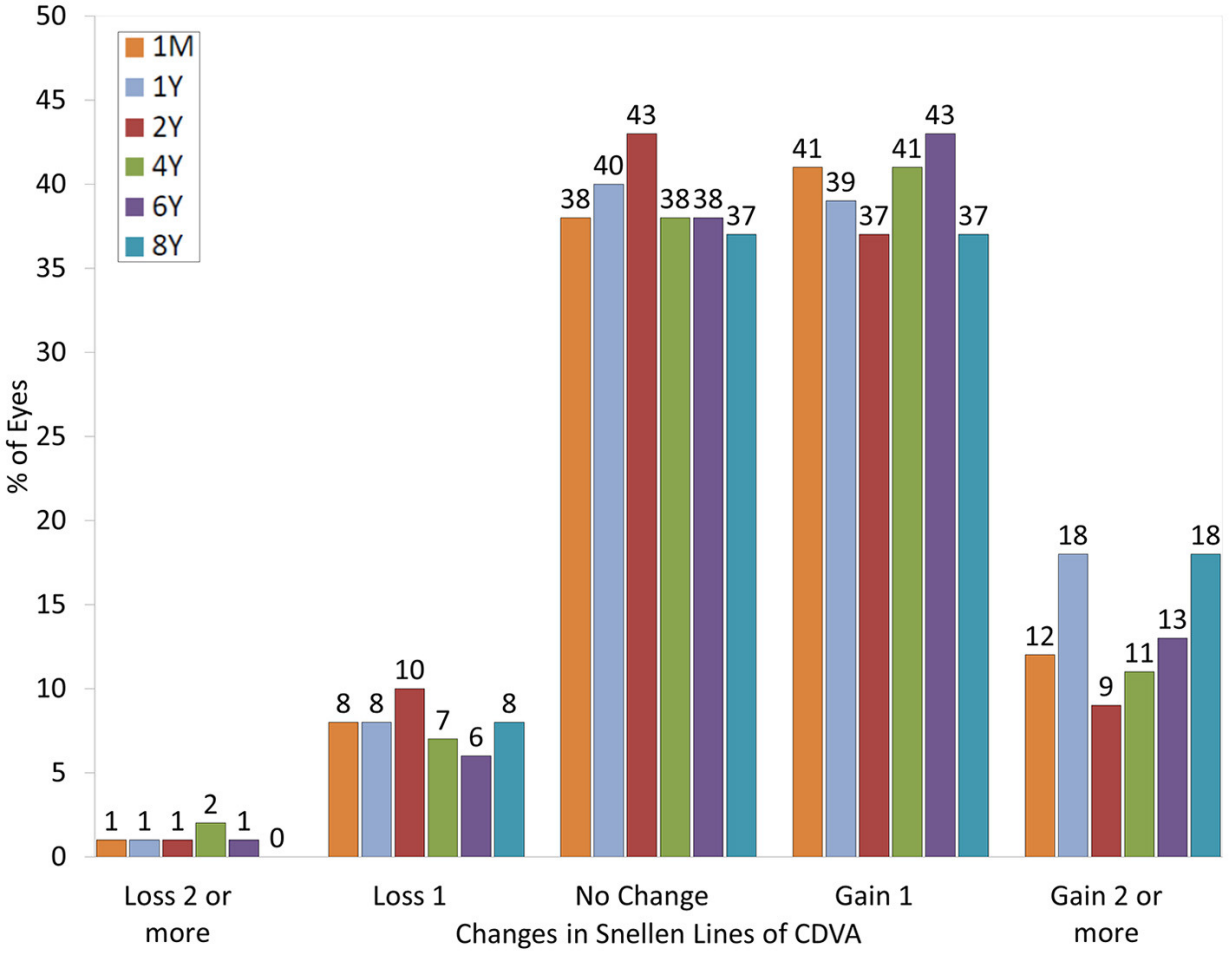
Changes in corrected distance visual acuity (CDVA) after hole implantable collamer lens (EVO-ICL) implantation.

At 4 years postoperatively, 68 eyes (38%) showed no change in CDVA, 73 eyes (41%) gained 1 line, and 20 eyes (11%) gained 2 lines, while 12 eyes (7%) lost 1 line and 4 eyes (2%) lost 2 lines. At 6 years postoperatively, 41 eyes (38%) showed no change in CDVA, 47 eyes (43%) gained 1 line, and 14 eyes (13%) gained 2 lines, while 6 eyes (6%) lost 1 line and 1 eye (1%) lost 2 lines. At 8 years postoperatively, 22 eyes (37%) showed no change in CDVA, 22 eyes (37%) gained 1 line, and 11 eyes (18%) gained 2 lines, while 5 eyes (8%) lost 1 line, but none of the eyes lost 2 lines or more (Figure 1). LogMAR CDVA was −0.21 ± 0.08, −0.21 ± 0.08, −0.20 ± 0.08, −0.21 ± 0.09, −0.21 ± 0.09, and −0.20 ± 0.09, respectively, at 1 month, and 1, 2, 4, 6, and 8 years postoperatively. We found a significant difference between preoperative CDVA and 4-, 6-, and 8-year postoperative CDVAs (*p* < 0.001, *p* < 0.001, and *p* = 0.002, respectively). The safety index (postoperative CDVA/preoperative CDVA) was 1.16 ± 0.24, 1.18 ± 0.26, and 1.18 ± 0.24, respectively, at 4, 6, and 8 years postoperatively.

**Figure 2:**
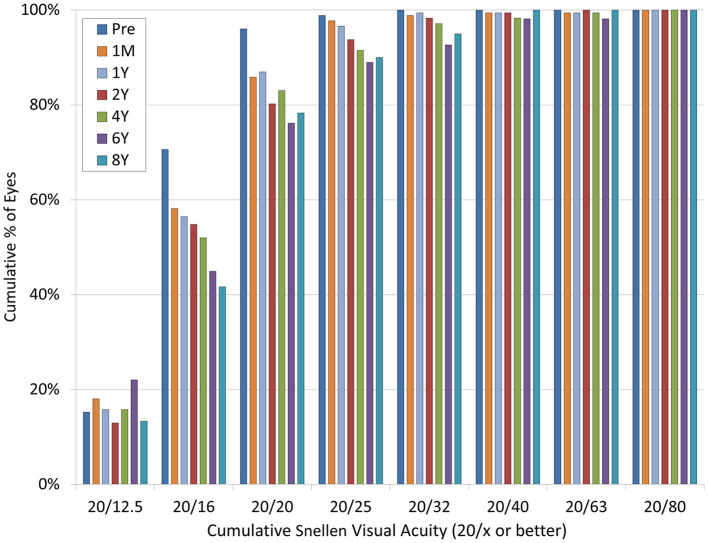
Changes in uncorrected distance visual acuity (UDVA) after hole implantable collamer lens (EVO-ICL) implantation.

At 1 month, and 1, 2, 4, 6, and 8 years postoperatively, 96, 86, 87, 80, 83, 76, and 78% of eyes, and 100, 99, 99, 99, 98, 98, and 100% of eyes, respectively, had a UDVA of 20/20 and 20/40 or better (Figure 2). LogMAR UDVA was −0.12 ± 0.14, −0.12 ± 0.14, −0.10 ± 0.14, −0.09 ± 0.17, −0.08 ± 0.21, and −0.07 ± 0.17, respectively, at 1 month, and 1, 2, 4, 6, and 8 years postoperatively. We found a significant difference between preoperative UDVA and 4-, 6-, and 8-year postoperative UDVAs (*p* < 0.001). The efficacy index (postoperative UDVA/preoperative CDVA) was 0.92 ± 0.29, 0.90 ± 0.31, and 0.89 ± 0.28, respectively, at 4, 6, and 8 years postoperatively.

### Predictability and Stability Outcomes

**Figure 3:**
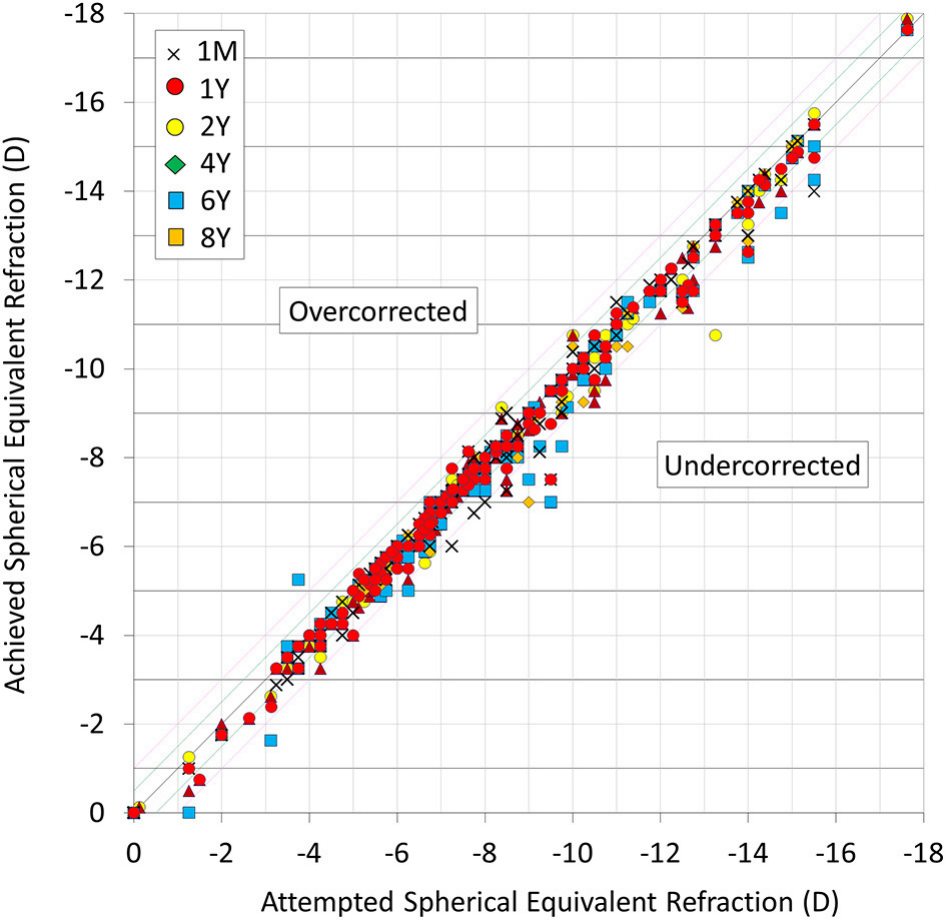
Scatter plot of attempted vs. achieved correction (spherical equivalent) after hole implantable collamer lens (EVO-ICL) implantation.

A scatter plot of the attempted vs. the achieved MSE correction is shown in Figure 3. At 1 month, and 1, 2, 4, 6, and 8 years postoperatively, 89, 90, 96, 92, 77, and 83% of eyes, and 97, 99, 99, 98, 90, and 93% of eyes were within ± 0.5 and 1.0 D, respectively, of the attempted spherical equivalent correction.

**Figure 4:**
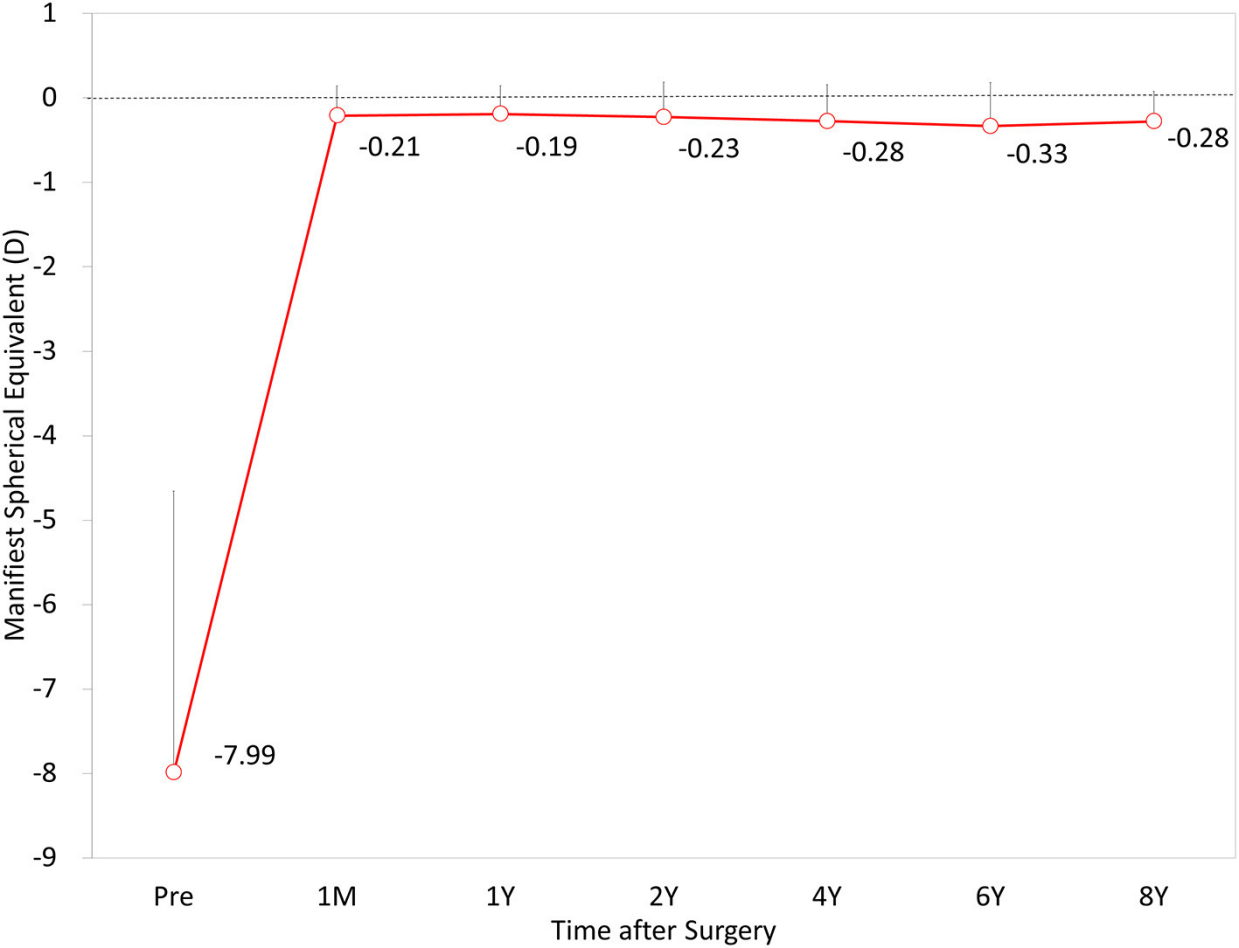
Time course of manifest spherical equivalent (MSE) after hole implantable collamer lens (EVO-ICL) implantation.

The time-course change in the MSE is shown in Figure 4. Preoperatively, and at 1 month, and 1, 2, 4, 6, and 8 years postoperatively, the MSE was −7.99 ± 3.33, −0.21 ± 0.35, −0.19 ± 0.33, −0.23 ± 0.42, −0.28 ± 0.43, −0.33 ± 0.51, and −0.28 ± 0.36 D, respectively (one-way ANOVA, *p* < 0.001). Changes in MSE refraction from 1 month to 8 years postoperatively were −0.13 ± 0.30 D.

### Intraocular Pressure

**Figure 5:**
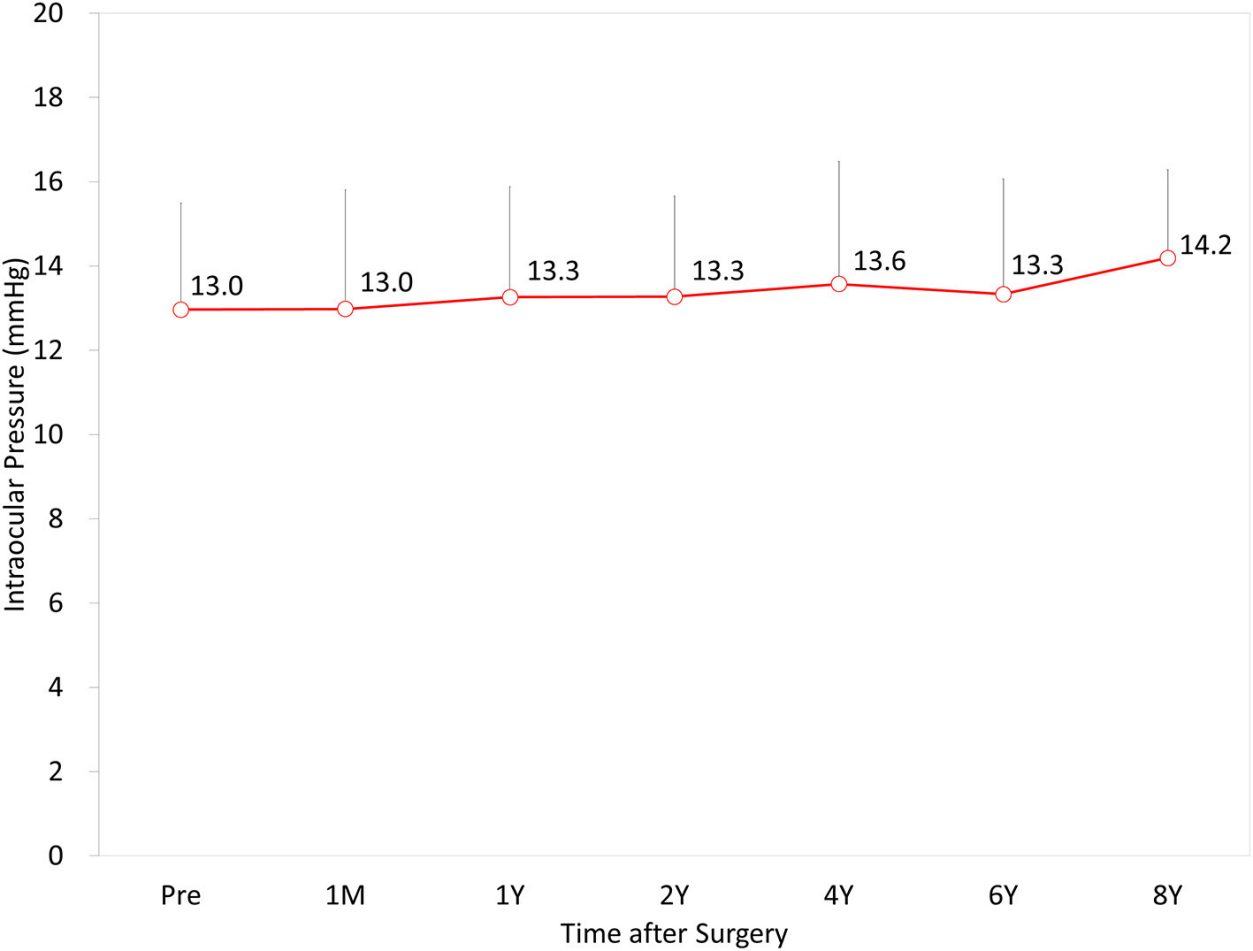
Time course of intraocular pressure (IOP) after hole implantable collamer lens (EVO-ICL) implantation.

The IOP was 13.0 ± 2.5, 13.4 ± 2.5, 13.3 ± 2.5, 13.6 ± 2.8, 13.5 ± 2.7, and 14.2 ± 2.1 mmHg, preoperatively, and at 1 month, and 1, 2, 4, 6, and 8 years postoperatively, respectively (*p* = 0.101) (Figure 5). No significant increase in the IOP (>25 mmHg) occurred in any case during the 8-year observation period.

### Endothelial Cell Density

The ECD significantly decreased from 2814 ± 231 cells/mm^2^ preoperatively to 2672 ± 237 cells/mm^2^ at 8 years postoperatively (*p* < 0.001). The mean percentage of the ECD loss was 3.6 ± 7.0 % at 8 years postoperatively.

### Vault

**Figure 6:**
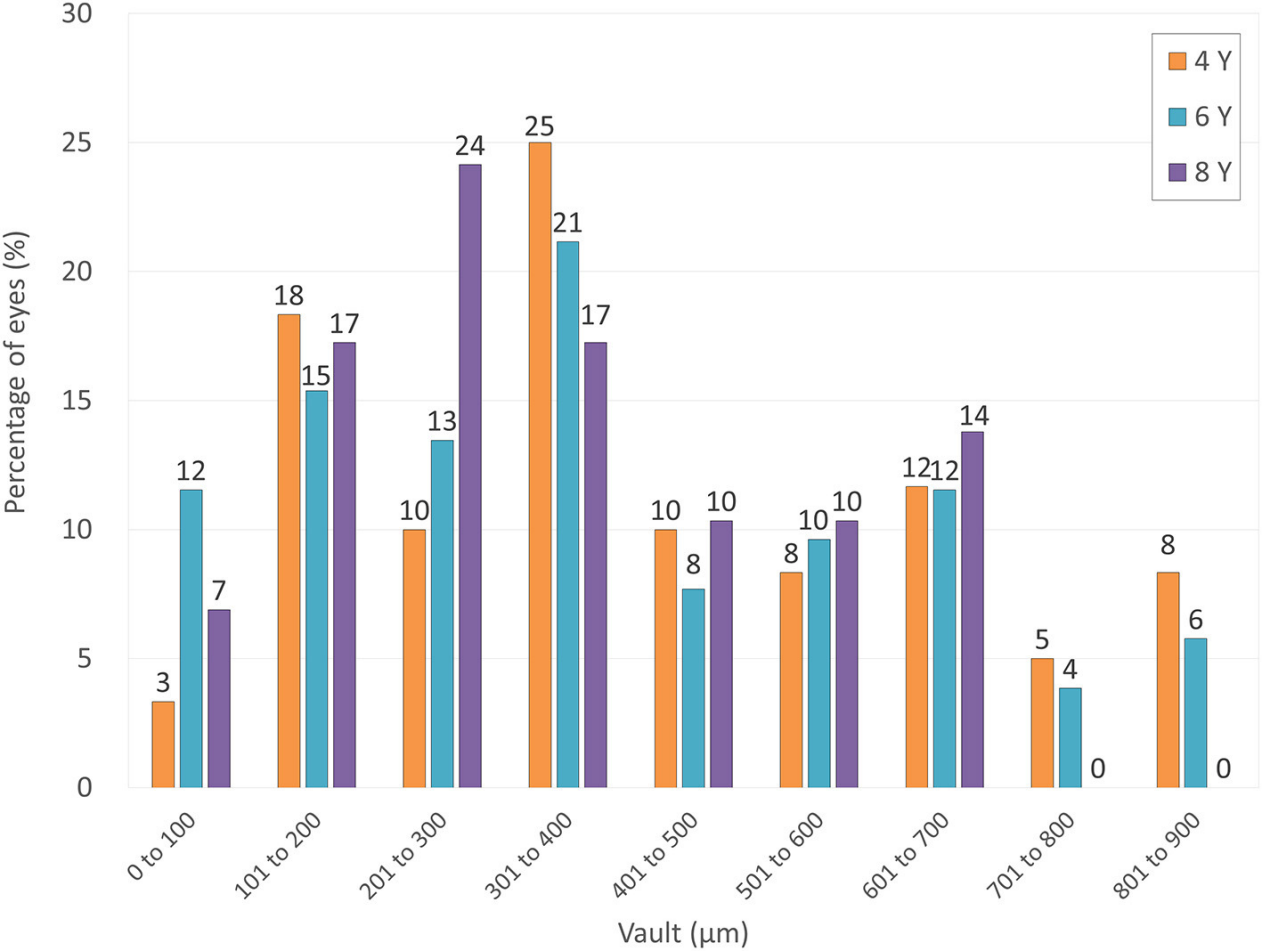
Distribution of eyes according to the vault at 4, 6, and 8 years after hole implantable collamer lens (EVO-ICL) implantation.

The ICL vault was 424 ± 227, 376 ± 225, and 347 ± 181 μm, at 4, 6, and 8 years postoperatively, respectively (*p* = 0.250). Figure 6 shows the postoperative distribution of the ICL vault. Neither excessive low vault (<50 μm) nor excessive-high vault (>1000 μm) requiring ICL exchange was found in any case throughout the observation period.

### Secondary Surgeries/Adverse Events

In this series, all surgeries were uneventful, and no apparent intraoperative complications such as an upside-down ICL insertion or traumatic cataract formation occurred. Table 2 summarizes the ICL-related and ICL-unrelated postoperative complications as well as the secondary surgical interventions throughout the 8-year follow-up. Two eyes (1.1%) required ICL repositioning at 1 week postoperatively (a 90° rotation to vertical orientation due to a high non-toric ICL vault and a re-alignment due to a toric ICL rotation). Two eyes (1.1%) required simultaneous ICL extraction and cataract surgery at 2 and 3 years postoperatively due to anterior subcapsular cataract formation. One eye (0.6%) required ICL size change (1 size up) at 7 years postoperatively to prevent peripheral attachment between the ICL and the crystalline lens and the subsequent progression of a peripheral anterior subcapsular cataract. All three eyes (1.7%) that developed cataracts had a slight pre-existing peripheral anterior subcapsular cataract formation preoperatively, and the UDVA at the final follow-up was 20/20 or more in these eyes. Two eyes (1.1%) required photorefractive keratectomy at 8 years postoperatively due to myopic regression. Otherwise, we found no significant complications, such as substantial IOP rise (>25 mmHg), pigment dispersion glaucoma, pupillary block, consequential ECD loss (>20%), severe subjective symptoms such as glare or halo, or other vision-threatening complications, during the observation period in this series.

**Table 2:** Postoperative complications and secondary surgical interventions after hole implantable collamer lens (EVO-ICL) implantation.

**Case**	**Age (years)**	**Gender**	**MSE (D)**	**ICL type**	**Onset**	**Complications**	**Intervention**
**ICL-related complications**							
Case 1	29	Male	−6.00	Non–toric	1 week	Excessive high vault	ICL rotation (horizontal to vertical plane)
Case 2	27	Female	−5.63	Toric	1 week	ICL rotation	ICL repositioning
Case 3	53	Female	−6.88	Non-toric	2 years	Anterior subcapsular cataract	ICL extraction and cataract surgery
Case 4	39	Female	−15.5	Non-toric	4 years	Anterior subcapsular cataract	ICL extraction and cataract surgery
Case 5	30	Male	−10.25	Non-toric	8 years	Myopic regression	Photorefractive keratectomy
	30	Male	−11.75	Non-toric	8 years	Myopic regression	Photorefractive keratectomy
Case 6	35	Male	−17.63	Toric	8 years	Anterior subcapsular cataract	ICL size change (1 size up)
**ICL-unrelated complications**							
Case 7	44	Male	−5.13	Non-toric	1 years	Retinal tear	Photocoagulation
Case 8	43	Male	−6.63	Toric	4 years	Epiretinal membrane	Observation

*MSE, manifest spherical equivalent; D, Diopter; ICL, implantable collamer lens*.

## Discussion

In the present study, our results demonstrated good long-term safety, efficacy, predictability, and stability outcomes during the 8-year observation period. Our results also confirmed that neither a significant IOP rise nor a significant ECD loss occurred in any case. In addition, it was observed that the current EVO-ICL implantation primarily decreased the onset of cataract formation compared to the conventional ICL implantation even at 8 years postoperatively. A total of three eyes (1.7%) developed cataracts in the current study, but it should be noted that all the eyes had a slight pre-existing peripheral anterior subcapsular cataract formation preoperatively, and the visual prognosis of such eyes was still good. Our findings indicate that this surgical procedure is one of the viable options for correcting moderate to high ametropia over a long period. To the best of our knowledge, this is the longest-term (spanning up to 8 years) study on the clinical outcomes of EVO-ICL implantation. We believe that this information will be clinically helpful for surgeons and patients for understanding the long-term prognosis of the current ICL.

Table 3 summarizes the long-term visual and refractive outcomes of conventional ICL implantation without a central port, spanning 10 years or more, and EVO-ICL implantation, spanning 5 years or more, for the myopic study population. Concerning the conventional ICL without a central port, Moya et al. (1) stated that the spherical equivalent refraction was −1.77 ± 1.93 D at 12 years postoperatively and that CDVA at the last visit was 0.22 ± 0.22 logMAR in 144 eyes undergoing ICL surgery. They also found that the incidence of clinically relevant cataracts was 13.88%, which was significantly linked to the use of the V3 model ICL. Guber et al. (2) reported that the rate of lens opacity development was 54.8% at 10 years postoperatively, owing to the broader view under full dilation and detection of peripheral lens opacity, but there was no significant increase in IOP observed during the 10-year follow-up. Kocová et al. (3) showed that the mean decimal UDVA and CDVA were 1.0 ± 0.37 and 1.18 ± 0.38 in myopic eyes, respectively, and 12.5% of myopic eyes developed cataracts, which significantly affected visual acuity. Nakamura et al. (4) demonstrated that logMAR UDVA and CDVA were −0.01 ± 0.24 and −0.18 ± 0.07 and that 71.4 and 87.1% of eyes were within 0.5 and 1.0 D, respectively, at 10 years postoperatively. They also found that the ECD loss was 5.3% at 10 years and 10.5% of 144 eyes developed anterior subcapsular cataracts during the 5- to 10-year follow-up period. Choi et al. (5) described no significant changes in the ECD or the IOP at any time point and reported that 12.1% of eyes developed lens opacities during the 10-year follow-up. We (6) also previously reported that the safety and efficacy indices were respectively 1.13 ± 0.27 and 0.83 ± 0.36, and that 68.3 and 85.4% of the eyes were respectively within 0.5 and 1.0 D of the targeted correction at 8 years postoperatively. Concerning the EVO-ICL, there have been only a few studies on the long-term (spanning 5 years or more) outcomes of this new technology-based surgery (12–14). Shimizu et al. (12) first showed, in a pilot study on 32 EVO-ICL-implanted eyes, that logMAR CDVA and UDVA were −0.24 ± 0.08 and −0.17 ± 0.14, respectively, at 5 years postoperatively, and that 96% of the eyes were within 1.0 D of the targeted correction at 5 years postoperatively. Alfonso et al. (13) described that logMAR CDVA and UDVA were respectively 0.02 ± 0.08 and 0.05 ± 0.11, at 5 years postoperatively, and that 67.4 and 90.1% of the eyes were within 0.5 and 1.0 D of the targeted correction, respectively. In a retrospective review of 84 EVO-ICL-implanted eyes, Fernández-Vega-Cueto et al. (14) recently demonstrated that logMAR CDVA and UDVA were 0.02 ± 0.08 and 0.17 ± 0.23, respectively, at 7 years postoperatively, and 53.57 and 80.95% of the eyes were within 0.5 and 1.0 D of the targeted correction, respectively. Interestingly, these long-term (spanning 5 years or more) studies confirmed no incidence of cataract formation after EVO-ICL implantation. Packer et al. (20) reviewed a total of 11 publications, including data on 617 eyes with a weighted average follow-up of 13 months, and reported a 0.49% incidence of asymptomatic anterior subcapsular cataract formation. Although we accept that several background factors, such as patient age, preoperative refraction, type of ICL, surgeon's experience, examiner's skill, and follow-up duration, could play a role in these surgical outcomes, we should be aware that the visual and refractive results were comparable with previous studies on conventional ICL implantation without a central port. However, it should also be noted that the onset of cataract formation has decreased mainly by the introduction of the new technology, possibly due to the improvement of the circulation of the aqueous humor to the anterior surface of the crystalline lens (7, 8).

**Table 3:** Summary for long-term outcomes of conventional implantable collamer lens implantation (spanning 10 years or more) and hole implantable collamer lens implantation (5 years or more) for myopia.

**Author**	**Year**	**Type**	**Period (years)**	**Eyes**	**Age (years)**	**MSE (D)**	**logMAR UDVA**	**logMAR CDVA**	**within ±1.0 D**	**Cataract**
Moya et al. (1)	2015	ICL V3, V4 (non-hole)	12	110	30.69 ± 5.59	−16.90 ± 4.26	0.49 ± 0.25	0.22 ± 0.25	34.3%	13.88%
Guber et al. (2)	2016	ICL V4 (non-hole)	10	75	38.8 ± 9.2	−11.4 ± 2.9	0.6, decimal	1.0, decimal	65.7%	54.8%
Kocová et al. (3)	2017	ICL V4 (non-hole)	10	40	28.28 ± 6.25	−11.0 ± 4.45	1.0, decimal	1.18, decimal	75.0%	12.5%
Nakamura et al. (4)	2019	ICL V4 (non-hole)	10	70	36.2 ± 7.7	−9.97 ± 2.29	−0.01 ± 0.24	−0.18 ± 0.07	87.1%	10.5%
Choi et al. (5)	2019	ICL V4 (non-hole)	10	110	30.3 ± 8.3	−12.01 ± 3.70	0.13 ± 0.20	0.03 ± 0.07	N.A.	12.1%
Shimizu et al. (12)	2016	ICL V4c (hole)	5	32	31.9 ± 7.5	−7.54 ± 2.40	−0.17 ± 0.14	−0.24 ± 0.08	100%	0%
Alfonso et al. (13)	2019	ICL V4c (hole)	5	147	31.24 ± 5.4	−9.20 ± 3.02	0.02 ± 0.08	0.02 ± 0.09	90.1%	0%
Fernández-Vega-Cueto et al. (14)	2021	ICL V4c (hole)	7	84	31.04 ± 4.89	−9.35 ± 2.90	0.17 ± 0.23	0.02 ± 0.08	100%	0%
Current	2021	ICL V4c, V5 (hole)	8	177	35.9 ± 7.9	−7.99 ± 3.33	−0.07 ± 0.17	−0.20 ± 0.09	93%	1.7%

*ICL, implantable collamer lens; MSE, manifest spherical equivalent; D, diopter; logMAR, logarithm of the minimal angle of resolution; UDVA, uncorrected distance visual acuity; CDVA, corrected distance visual acuity*.

There are several limitations to this study. Firstly, this research was conducted in a retrospective fashion. Indeed, some patients dropped out of the routine follow-up. Considering that visually satisfied patients were lost during the follow-up, we speculate that the actual outcomes could be better than these outcomes. However, this review of the clinical charts may reflect the actual status of hospital-based ICL surgery in a clinical setting. Secondly, the number of patients who completed the 8-year observation were rather limited. A prospective study with a large number of patients would be ideal for accurately grasping the long-term outcomes of this new surgical procedure. Moreover, we did not quantitatively assess the amount of the ICL vault in the early stages of the present study since we introduced the AS-OCT in our hospital in 2017. Further research on the stored ICL vault data using the AS-OCT may provide helpful information to assess the long-term changes in the ICL vault in the future.

## Conclusions

In summary, our long-term outcomes confirmed that ICL implantation provided good results in terms of long-term safety, efficacy, predictability, and stability, and vision-threatening complications did not occur in any case during the 4- to 8-year observation period. Although the visual and refractive outcomes of EVO-ICL implantation were almost comparable with those of the conventional ICL implantation, we emphasize that late-onset cataract formation, which was one of the concerns about ICL surgery, decreased considerably due to the introduction of the central port technology. Thus, our findings support the view that current ICL implantation with a central port is one of the viable surgical options for correcting moderate to high ametropia over a long period.

## Data Availability Statement

The original contributions presented in the study are included in the article/supplementary material, further inquiries can be directed to the corresponding author/s.

## Ethics Statement

The studies involving human participants were reviewed and approved by Institutional Review Board at Kitasato University Hospital (identifier: B21-118). Written informed consent for participation was not required for this study in accordance with the national legislation and the institutional requirements.

## Author Contributions

KK, KS, and NS were involved in the design and conduct of the study. MT, WA, and HH were involved in collection, management, analysis, and interpretation of data. KK, KS, WA, HH, and NS were involved in the preparation, review, and final approval of the manuscript.

## Conflict of Interest

KS was a paid consultant for STAAR Surgical. The remaining authors declare that the research was conducted in the absence of any commercial or financial relationships that could be construed as a potential conflict of interest.

## Publisher's Note

All claims expressed in this article are solely those of the authors and do not necessarily represent those of their affiliated organizations, or those of the publisher, the editors and the reviewers. Any product that may be evaluated in this article, or claim that may be made by its manufacturer, is not guaranteed or endorsed by the publisher.
